# Genome Sequences of Two Tunisian Field Strains of Avian *Mycoplasma*, *M. meleagridis* and *M. gallinarum*

**DOI:** 10.1128/genomeA.00558-16

**Published:** 2016-06-16

**Authors:** Elhem Yacoub, Pascal Sirand-Pugnet, Aurélien Barré, Alain Blanchard, Christophe Hubert, Florence Maurier, Emmanuel Bouilhol, Boutheina Ben Abdelmoumen Mardassi

**Affiliations:** a Unit of Mycoplasmas, Laboratory of Molecular Microbiology, Vaccinology and Biotechnology Development, Institut Pasteur de Tunis, University of Tunis El Manar, Tunisia; b INRA, UMR 1332 de Biologie du Fruit et Pathologie, Villenave d’Ornon, France; c Université of Bordeaux, UMR 1332 de Biologie du Fruit et Pathologie, Villenave d’Ornon, France; d Centre de Bioinformatique et de Génomique Fonctionnelle, CBiB, Université of Bordeaux, Bordeaux, France; e Plateforme Génome-Transcritpome de Bordeaux, CGFB, Université of Bordeaux, Bordeaux, France

## Abstract

*Mycoplasma meleagridis* and *Mycoplasma gallinarum* are bacteria that affect birds, but little is known about the genetic basis of their interaction with chickens and other poultry. Here, we sequenced the genomes of *M. meleagridis* strain MM_26B8_IPT and *M. gallinarum* strain Mgn_IPT, both isolated from chickens showing respiratory symptoms, poor growth, reduction in hatchability, and loss of production.

## GENOME ANNOUNCEMENT

*Mycoplasma meleagridis* and *Mycoplasma gallinarum* belong to the hominis phylogenetic group within the *Mollicutes* class. Turkey was considered to be, for a long time, the specific host for *M. meleagridis* (1), until its isolation from chickens was reported (2). The ubiquitous nature of *M. gallinarum* in poultry as well as mammals has been reported by many investigators (3). It is considered to have a commensal relationship with its hosts. In poultry, *M. gallinarum* is commonly thought to be unable to cause disease by itself. Currently, the only available information that can provide us with data on genetic factors that might explain the host tropism and pathogenic potential of *M. meleagridis* and *M*. *gallinarum* species is limited to the information given by the genome sequences of *M. meleagridis* type strain ATCC 17529 (4), *M. meleagridis* Italian field strain (5), and *M. gallinarum* field strain DSM 19816 (GenBank accession no. JHZE00000000).

To facilitate our understanding of the host tropism and pathogenic potential of *M. meleagridis* and *M. gallinarum*, we sequenced the genomes of two field strains of *M. meleagridis* MM_26B8_IPT and *M. gallinarum* Mgn_IPT.

Both strains were isolated from chicken farms located in the Cap Bon region (northeast) of Tunisia. Strains were cultivated in Frey’s medium (6) and purified three times by filter cloning. Identification was confirmed by sequencing the PCR products of the 16S rRNA genes.

The MM_26B8_IPT and Mgn_IPT genomes were sequenced, annotated, and analyzed as described previously (4). Briefly, sequencing was achieved by combining mate-pair and paired-end Illumina MiSeq technologies. High-quality filtered reads were assembled using CLC and ABySS workbenches. The annotation was automatically performed by RAST/SEED server (7) and manually improved after implementation into the MolliGen database (8). The Aragorn software (9) was used to recognize tRNA and transfer-messenger RNA (tmRNA) genes. rRNA genes were located on the genomes using the RNAmmer 2.1 software (10).

The MM_26B8_IPT genome has a size of 658,083 bp (assembled in 32 contigs), whereas the Mgn_IPT one is smaller, consisting of 800,663 bp (organized in 56 contigs). The G+C content in the two genomes was almost the same, around 26%. A total of 539 and 601 coding sequences (CDSs) were identified in MM_26B8_IPT and Mgn_IPT, respectively. The more the genome was reduced, the greater was the coding density (90.44% in MM_26B8_IPT versus 87.40% in Mgn_IPT). Both strains were found to contain 33 tRNAs and a single copy of tmRNA.

Among the 9 and 10 genes involved in the stress response determined in MM_26B8_IPT and Mgn_IPT, respectively, 8 heat shock proteins (DnaK, DnaJ, GrpE, HrcA, LepA, SmpB, and 2 rRNA methyltransferase subunits) were shared. In addition, 17 and 14 genes potentially implicated in virulence and resistance to antibiotics and toxic compounds were identified in MM_26B8_IPT and Mgn_IPT, respectively.

Further analyses of the genome sequences of these two avian field strains, *M. meleagridis* and *M. gallinarum*, isolated from Tunisian chickens will help further the study of their host specificity and pathogenic potential.

### Nucleotide sequence accession numbers.

These whole-genome shotgun projects of *M. meleagridis* and *M. gallinarum* field strains have been deposited at DDBJ/EMBL/GenBank under the accession numbers LVWO00000000 and LVLH00000000, respectively. The versions described in this paper are versions LVWO00000001 for *M. meleagridis* MM_26B8_IPT and LVLH00000001 for *M. gallinarum* Mgn_IPT.
